# Data Analysis and Modelling of Billets Features in Steel Industry

**DOI:** 10.3390/s22197333

**Published:** 2022-09-27

**Authors:** Silvia Maria Zanoli, Crescenzo Pepe, Elena Moscoloni, Giacomo Astolfi

**Affiliations:** 1 Dipartimento di Ingegneria dell’Informazione, Università Politecnica delle Marche, Via Brecce Bianche 12, 60131 Ancona, Italy; 2 Alperia Green Future, 60015 Falconara Marittima, Italy

**Keywords:** steel industry, reheating furnace, rolling mill stands, billet, tracking system, data analysis, modelization

## Abstract

This study proposes a data analysis and modelization method for the rolling mill process of billets in steel plants. By exploiting rolling mill signals and advanced data processing algorithms, a reliable billet tracking system is designed, which tracks each workpiece from the furnace entrance to the rolling mill stands’ exit area. Based on the stored information, two problems are addressed: the data analysis of the temperature sensors (a thermal imaging camera and pyrometers) and the current that is related to the rolling mill stands’ absorption, and subsequently, a mathematical modelization of the billets’ temperature along their path in the rolling mill is produced. The data analysis suggested that we should perform hardware modifications: the thermal imaging camera was repositioned to avoid the effect of scale formation on the temperature measurements. The modelization phase provided the basis for future control and/or diagnosis applications that will exploit a temperature decay model.

## 1. Introduction

The secondary sector exploits a lot of energy all over the world. Metallurgical, petrochemical, textile, cement, and food industries are examples of energy-consuming processes [1,2]. Green Economy energy policies have attracted the attention of engineers and researchers; in order to contain and mitigate the environmental risks that energy-consuming processes pose, smart and preventive actions have to be designed and implemented [3]. In this context, additional programs have been introduced in the last few years, e.g., Agenda 2030 [4] at the global level and PNRR (Piano Nazionale di Ripresa e Resilienza) at the Italian level [5].

Among the metallurgical industries, the steel industry is one that is of primary importance in the national and international contexts. In the steel industries, many different production phases can be identified, which require a lot of energy (e.g., electrical and thermal). In order to reduce the quantity of CO_2_ emissions, different approaches can be exploited [6]. CO_2_ emission reduction is a challenging objective that can be achieved, for example, by carrying out hardware modifications to the plant and/or exploiting a set of methods that do not necessarily require that the plant changes [7]. These methods are based on data analysis, modelization techniques, and control/optimization algorithms [8,9,10].

In the steel industry, the reheating of the furnaces and the rolling mill stand use phases play a fundamental role in terms of energy consumption and the quality of the product. Semi-finished products, e.g., billets, are reheated in the reheating furnaces and then they are plastically deformed in the rolling mill stands. The specifications of the whole process depend on the type of defined final product that is produced, e.g., rods and bars [11,12,13,14,15,16]. This study proposes a data analysis and modelization method for the rolling mill process of billets in steel plants.

Reheating and rolling processes require advanced monitoring, control, and optimization systems in order to save energy while preserving the product quality. Real-time information of the billets’ temperatures while they are inside the furnace is not available [17,18]. Different types of measurements can be associated with processed billets online: all of the data that are available in the considered process (e.g., triggers, photocells, temperature measurements, and absorption measurements) need to be carefully analyzed, i.e., accurate relationships and correlations are a necessary requisite for monitoring, modeling, control, and optimization. 

With regard to conducting data analysis and modelization in the steel industry, different approaches have been proposed in the literature at different levels of automation hierarchy and for each different subprocess. In [19], multivariate time-series data from a steel manufacturing industry are considered and a generative adversarial network (GAN)-based data imputation technique is proposed. An abnormal data detection method that is based on a multi-criteria evaluation is proposed in [20], which focuses on energy data. An AFCM (Adaptive fuzzy C-means) algorithm is used for the outlier detection. In [21], data analysis is exploited for modelization purposes by analyzing a large number of monitoring data of the rolling mill condition and by using a neural network model. In [22], a smart steel manufacturing framework is proposed in an Industry 4.0 context: an automatic identification tracking method for steel products is developed that is based on a vision-based approach. In [23], the Data Mining concept that is used to processes technological data is applied to steel billet heating in a continuous furnace of a rolling-mill shop. A regression dependence of the steel billet temperature that is based on its furnace heating history is obtained. In [24], a mathematical model of the billet heating process in a regenerative reheating furnace is established to predict the state of the billets in the furnace after a period of delay rolling. An adaptive genetic algorithm and a particle swarm optimization algorithm are used, and the results of these are compared. In [25], a prediction model that is based on a gate recurrent unit was established to forecast the inside temperature of the furnace by measuring the temperature, fuel, and air-time series. In [26], a finite-difference model of a steel heating process is developed using differential equations of transient heat conduction.

To the best of the authors’ knowledge, a critical data analysis on the values that are measured through different sensors in a rolling mill is not present in the literature. Furthermore, the relationship between the data analysis and the sensor location has also not been addressed in the literature. Modelization studies of the thermal behavior of billets in a rolling mill that are targeted at developing control/optimization algorithms have not been proposed in the literature. Reliable data allow researchers to obtain reliable models; reliable models represent the basis for robust control and optimization algorithms [1,2]. The achievement of reliable data and models represent the objectives of the present work. This paper extends the preliminary contents that are reported in [27]. An in-depth literature review is added, and the theoretical approach that is developed for billet tracking is described in detail, along with the process information and insights into data acquisition and analysis techniques. New field results and a more detailed discussion are provided.

In the following study, a data analysis and modelization method for the rolling mill process of billets in steel plants is proposed. A billet tracking system is designed in order to collect and store all of the fundamental data from the furnace entrance to the rolling mill stands’ exit area. Different plant configurations are considered. Based on the stored data, two problems are addressed: first, the analysis of the data that are acquired by different sensors (a thermal imaging camera, pyrometers, and the stands’ absorption) is carried out; then, a mathematical modelization of the billets’ temperature along their path in the rolling mill is proposed.

The papers is organized as follows: Section 2 reports the material and methods, providing the process description, details on the data selection, acquisition, storage, and analysis, the billet tracking system, the modelization, and the computational framework. Section 3 reports the results and discussion, focusing on the data analysis and the modelization. The conclusions are summarized in Section 4, together with some ideas for future work.

## 2. Materials and Methods

### 2.1. Process Description

**Figure 1:**
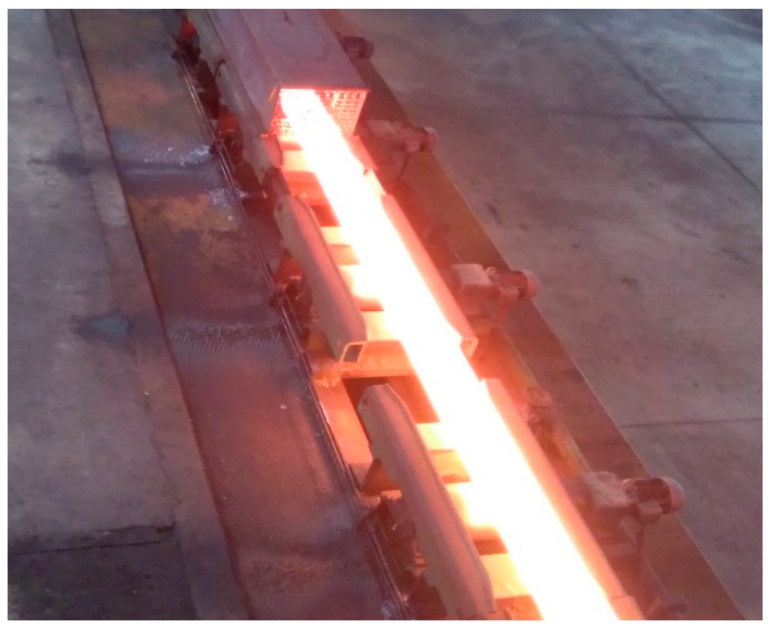
Example of a billet.

The present paper refers to the reheating furnaces and rolling mills that are located in steel plants. Billets are the workpieces that are processed in them (Figure 1). The billets that are processed in the considered plant have a size of 0.14 m × 0.14 m or 0.16 m × 0.16 m and are characterized by a 7–12 m length.

**Figure 2:**
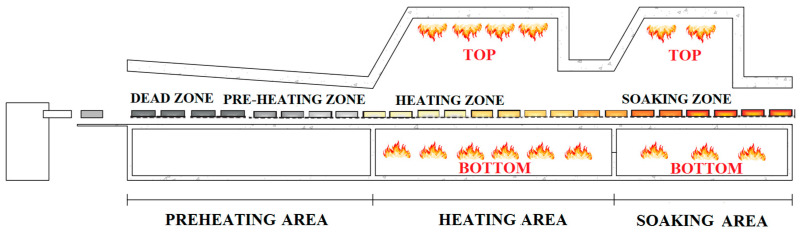
Schematic representation of the reheating furnace.

The reheating furnace that is considered as an example in this study is a walking beam reheating furnace [11,28]. The billets are introduced into the furnace through suitable pushers (front charging), and they are spaced apart from one another; their movement in the furnace is triggered by the following procedure: the billets are lifted and are moved forward (or backward) by a defined step. The step that is used mainly depends on the billets’ section. In this way, the billets are moved through the furnace and then they reach the rolling mill stands: here a hot rolling phase takes place (Figure 2).

The temperature of the upper layer of the billets is detected by an optical pyrometer that is located near the furnace’s entrance. The billets that are processed in the considered plant are mostly cold billets (at about 30 °C at the furnace inlet).

**Figure 3:**
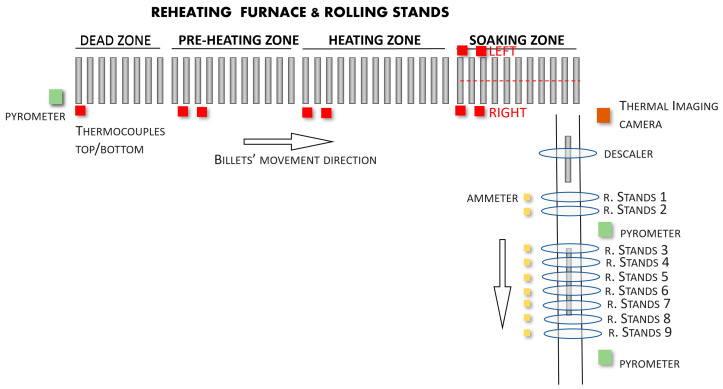
*Original* configuration of the plant.

As it can be noted in Figure 2, the billets enter the considered reheating furnace from the left side, but nearly the first half of the furnace is not equipped with active burners (Preheating Area). The Preheating Area is characterized by a dead zone and by a preheating zone that is not equipped with active burners. Near the Preheating Area, a chimney is present for smoke expulsion to occur. Immediately after they have left the Preheating Area, the billets move through the Heating Area. In the Heating Area, a crucial phase of billet heating takes place. Finally, the Soaking Area equalizes the billet heating process before their exit from the furnace. The equalization phase refers to the equalization of the heating that occurs between the head and the tail of the billets (right/left in the furnace configuration of Figure 3). This phase is critical within a reheating furnace because the head and tail of the billets may have to be heated differently to overcome the delay that occurs between the treatment of the billet head and tail within the rolling mill stands.As it can be noted in Figure 2, the billets’ temperature is subject to an increase during their period within the furnace: the billets’ reheating is depicted through the color scale that ranges from grey to red in Figure 2. The grey color represents the cold billets, i.e., the billets that are in the preheating phase; the yellow and orange colors represent the billets that are in the heating phase, while the red part is used for representing the billets that are in the soaking phase. Due to the furnace walking beam type that is used, empty spaces are present between the billets. Table 1 reports the furnace zone lengths.

Two thermocouples, which are positioned at the top and bottom areas of the furnace, measure the temperature of each furnace zone (not including the dead zone). In the Soaking Area, there are two firing zones (left and right); in each of the two zones, two thermocouples that are positioned at the top and bottom areas of the furnace are present. In order to monitor the temperature of the dead zone, the smoke exchanger thermocouple is used.

Active burners are located in the top and bottom Heating and Soaking Areas (see Figure 2). The Soaking Area, being characterized by two firing zones (left and right, see Figure 3), contains two independent sets of burners. Each furnace zone with burners is regulated through the Proportional-Integral-Derivative (PID) controllers with a master-slave setup between the top (master) and the bottom (slave) burners [29]. The PID setpoints are manipulated based on the furnace production rate, furnace inlet temperature, steel quality, and the final product that is to be obtained; the setpoints are adjusted in order to guarantee a furnace outlet–billet temperature of about 1200 °C. The PID setpoints are in the range 1080–1230 °C. Figure 3 shows a more detailed schematic of the reheating furnace and of the rolling mill stands. The configuration that is reported in Figure 3 will be denoted as the *original* configuration. All of the previously described sensors and furnace features are reported in Figure 3.

In Figure 3, at the exit area of the furnace (from the right side, with respect to the furnace longitudinal axis), a temperature sensor (a thermal camera) can be noted. This sensor detects the billets’ final temperature at the end of the first heating phase. From this point, the semi-finished products advance on the rollers up to a descaler device. The descaler device is an instrument, which, by means of employing high-pressure water jets, guarantees the removal of the scale or the impurities that are caused by the oxidation of the steel on the surface of the billets that is caused by the heating process. The presence of scale possibly causes problems on the measurements that are taken by the temperature sensors; the scale, in fact, is characterized by a temperature that is generally lower than that of semi-finished product itself [30,31].

After the descaler device is used, the billets are subjected to the rolling phase (Figure 3). In the considered rolling mill, the rolling phase is characterized by two sub-phases: in the first sub-phase, the billets continue their movement on the transport rollers, thereby reaching the first two rolling mill stands (stands 1-2, see Figure 3). After they have reached stands 1-2, first, the rolling mill optical pyrometer measures the billets’ temperature. Subsequently, the billets enter the second rolling mill area which is composed of stands 3-4-5-6-7-8-9 (seven stands in total, see Figure 3). At the end of the rolling mill stands, an additional optical pyrometer is located here (second rolling mill pyrometer, see Figure 3). The distance between the furnace’s exit and the last optical pyrometer is about 50 m, and billets are moved on the rolling mill transport rollers at an average movement speed of about 1 m/s.

The measuring ranges of the thermal imaging camera, the furnace inlet pyrometer, and the first/second rolling pyrometer are reported in Table 2. Suitable calibration tests of the different instruments were performed in order to ensure reliable data acquisition. In some cases, the tuning coefficients of the instruments were adjusted in order to match the measurements that were acquired using test devices, e.g., portable pyrometers.

**Figure 4:**
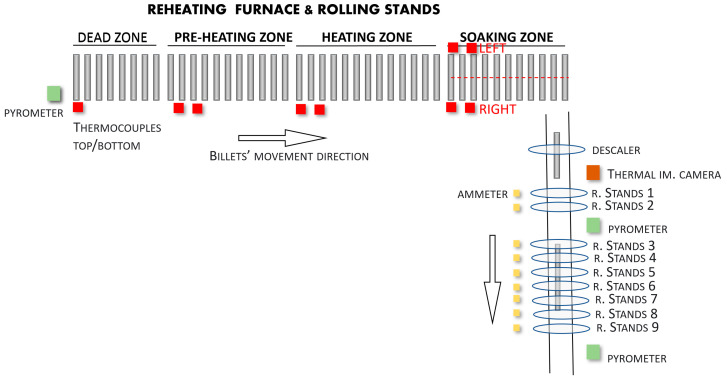
*Modified* configuration of the plant.

After the first analysis phase that is reported in the present paper was conducted, a *modified* configuration has been implemented in the field, in order to improve the data reliability in relation to the thermal imaging camera measurements, mainly due to the presence of the scale on the billet’s surface (see Section 3). Figure 4 reports the *modified* configuration that was used. The only difference between the *original* configuration (Figure 3) and the *modified* configuration (Figure 4) is the relocation of the thermal imaging camera: in the *original* configuration, the thermal imaging camera was located at the furnace’s exit, while in the modified configuration, it was moved to a position that is located after the descaler device and before the first rolling mill stand in the order of the configuration.

### 2.2. Data Selection, Acquisition, and Storage

With regard to the plant data, suitable selection, acquisition, and storage procedures have been adopted. The plant data were acquired in the field through the PLCs (Programmable Logic Controllers) [10]. First, an accurate data selection phase has been performed. Table 3 reports the selected data that are related to the billets, the reheating furnace, and the rolling mill that were selected for the present study [1].

**Figure 5:**
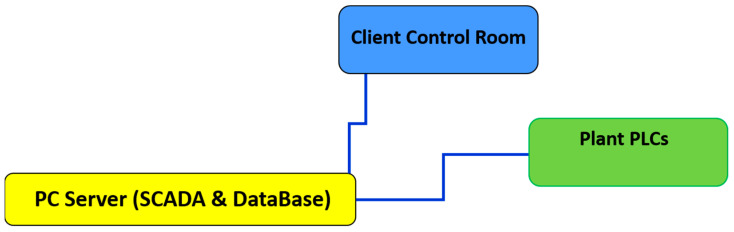
Designed configuration for data acquisition and storage.

In order to acquire and store the necessary data for the study, a suitable architecture has been designed (Figure 5). As previously described, the selected data were acquired from the plant through the PLCs (*Plant PLCs*, Figure 5). PLCs manage the lower levels of the automation hierarchy; they ensure the proper operation of the plant and its safety. A PC server has been installed in the plant, and it was connected to the plant’s net infrastructure. On the PC Server, a Supervisory Control And Data Acquisition (SCADA) system has been installed and a database has been created (*PC Server* (*SCADA and database*), Figure 5) [1,2,10]. The data selection, acquisition, and storage procedures were implemented on the PC server. Furthermore, a PC client has been installed in the control room of the reheating furnace in order to provide selected signal information to the plant operators and engineers. Tailored data visualization methods were designed in order to make the provided information user-friendly.

### 2.3. Billet Tracking System

In order to exploit the information that is stored in the created database, a billet tracking system has been developed. The system tracks each processed billet from the reheating furnace inlet to the rolling mill stands’ exit area; the billet tracking system, thanks to the acquired data and the plant information, takes into account the *original*/*modified* plant configuration (Figure 3 and Figure 4).

From the field sensors’ data inspection, acquisition errors and/or devices malfunctions are commonly observed. Suitable data preprocessing techniques (e.g., validity limits and spike and freezing checks) were applied in order to detect the bad data that were discarded. Validity limits and spike and freezing thresholds were tuned based on the sensor data sheets and the historical data. By exploiting reliable data, the output of the billet tracking system has been organized into a tailored structure. The structure is composed of 15 columns, and each line refers to the “history” of one processed billet. Billet features (e.g., mass, length, section, mass per length, and reheating group) are reported in first column. The reheating group establishes the reheating curve that should be achieved for the considered billet. The steel quality code is reported in the second column, together with the diameter of the finished product that is obtained after the rolling phase of the selected billet. The timestamp that is related to the time when the billet entered the furnace inlet is reported in the third column. The temperature values that are provided by the furnace inlet pyrometer that are related to the billet’s temperature at the furnace inlet are reported in the fourth column. The fifth column contains the discretization time-step sequence of the billet in the furnace. A matrix containing the thermocouple values that have tracked the considered billet, according to the fifth column, is reported in the sixth column. The structure that is described for the reheating furnace has been replicated for the rolling mill stand measurements, considering the billet’s furnace exit time, the ambient temperature, the thermal imaging camera, and the two rolling mill pyrometers, the stands’ absorption rate, and the associated discretization time stamps. Furthermore, additional information on the billets are achieved through the tracking system (e.g., if a billet is equipped with an iron wire tie that has to be removed by the operators at the furnace exit, thereby causing an abnormal residence time for the considered billet in the rolling mill stands, or if a billet has been reloaded into the furnace through the exit door due to unexpected events in the rolling mill stands).

### 2.4. Data Analysis

Based on the billet tracking system output, data analysis techniques were applied. In particular, correlation and regression concepts have been exploited [32,33,34].

The correlation coefficient and the determination index between the temperature measurements and stands’ absorptions values have been calculated for each billet that was recorded through the billet tracking system. Moreover, graphical results (scatter plots) were produced. The billet datasets, which are available for each production day, were initially used for a data analysis; subsequently, the datasets that contain the same days were divided according to different criteria to determine what was the most meaningful way to partition the information, i.e., the partition criteria that led to a notable improvement in the indices were the ones that were chosen. These partition criteria provided the work datasets for the evaluation of the analyzed plant configuration and for the mathematical model (see Section 2.5).

Assuming that a cluster can only be composed by billets that were produced on the same day, with the same geometry, and with comparable furnace inlet temperature ranges (e.g., hot or cold), the considered partition criteria of the billet datasets are:

- Billets that were processed during the same production day;
- The billet reheating group (1, 2, 3, and 4). Based on the plant information, 0.14 m × 0.14 m billets can only be associated with group 4;
- The thermal imaging camera temperature ranges: by considering the temperature that was measured by the thermal imaging camera, the billet datasets were suitably created. In this way, groups of billets that are homogeneous with respect to the temperatures that were detected at the furnace outlet were obtained;
- The diameter of the finished products;
- The steel quality: this partitioning method consisted of one sub-clustering of the previous criterion (diameter); the billets were further partitioned on the basis of their steel quality. The resulting clusters contain billets that are characterized by same quality as well as the same diameter.

With regard to the thermal imaging camera temperature range criterion, for each production day, the mean value and standard deviation of the thermal imaging camera measurements were calculated and with these, seven groups were created.

**Figure 6:**
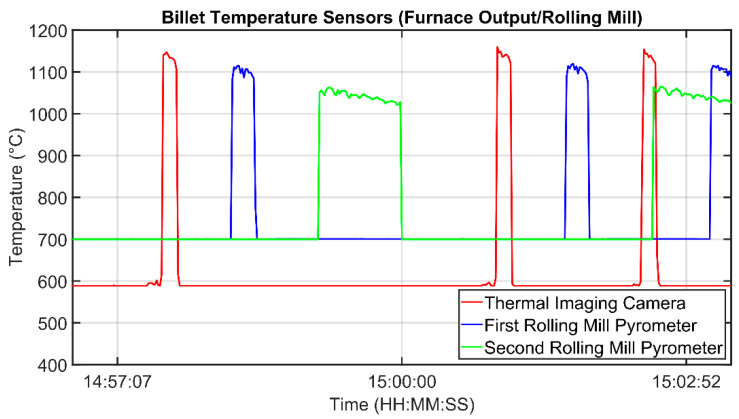
Billet temperature sensors measurements (furnace output/rolling mill).

**Figure 7:**
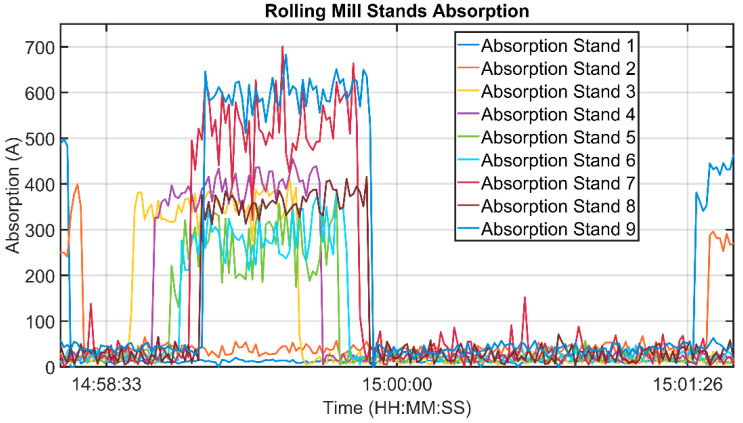
Rolling mill stands’ absorption measurements.

In order to calculate the determination index and the correlation coefficient between the temperature measurements and the stands’ absorptions values, all of the instruments have been considered: three temperature sensors (a thermal imaging camera, the first rolling mill pyrometer, and the second rolling mill pyrometer) and the absorptions measurements of nine stands (1–9, see Figure 3 and Figure 4). Because for each billet different measurement samples were available from each sensor, percentile-based algorithms were used in order to obtain a unique “global” value for each sensor for each tracked billet. Figure 6 and Figure 7 show the typical trends of the measurement that were provided by each sensor: the signal trends motivated the previously described procedure for the achievement of a unique “global” value for each sensor for each tracked billet. Figure 6 reports the thermal imaging camera (red), the first rolling mill pyrometer (blue), and the second rolling mill pyrometer (green) measurements. A billet that exits the furnace, moves, first, under the thermal imaging camera and subsequently, under the two pyrometers (see Figure 3 and Figure 4). As it can be noted in Figure 6, the temperature magnitude decreases with the progress of the billet path within the rolling mill stands. Figure 7 reports the current absorptions of the rolling mill stands with different colors. A billet that starts to be rolled is subjected, first, to processing which is conducted by stands 1 and 2 (light blue and orange signals, respectively, on the left side of Figure 7). Subsequently, according to the layout of the plant (see Figure 3 and Figure 4), several seconds elapse before the billet enters stand 3 (yellow signal), and then, the other five stands.

### 2.5. Rolling Mill Stands’ Billet Temperature Decay Mathematical Modelization

The designed rolling mill stands billet temperature decay mathematical model takes into account the path of the tracked billets from the furnace’s exit to the rolling mill stands’ exit areas. The start and end points of the model are defined by the passing of the billets in front of the thermal imaging camera and the second rolling mill pyrometer (downstream of stand 9). Note that, depending on the plant configuration that is under study (Figure 3 and Figure 4), the analyzed path can change. The model that has been used was based on heat transfer equations ([1,28]) and on a division of the billets’ path, which ran outside of the reheating furnace, as based on the plant’s configuration.

**Figure 8:**
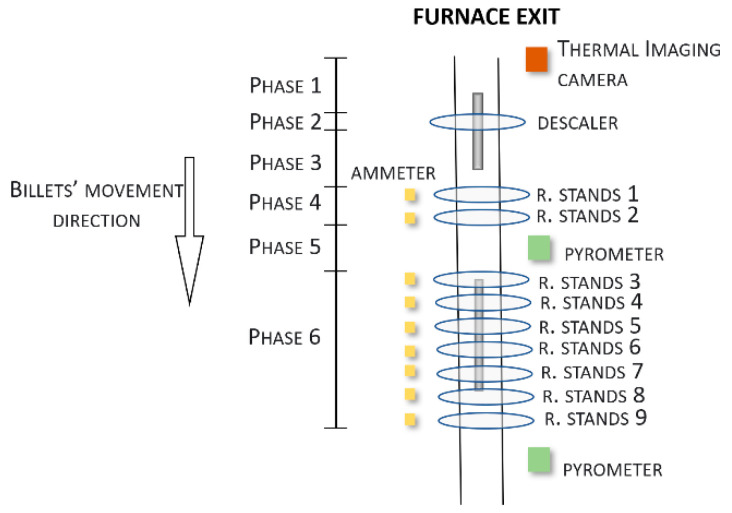
*Original* configuration of the plant (including details on the rolling mill phase).

Considering the *original* plant configuration (Figure 3 and Figure 8), a division of the billets’ path, which ran outside the reheating furnace, into six phases was proposed:

1. Thermal imaging camera-descaler movement;
2. Descaler processing;
3. Descaler-stand 1 movement;
4. Rolling mill stand 1–2 processing;
5. Stand 2-stand 3 movement;
6. Stand 3-stand 9 processing.

Figure 8 reports the details on each phase that is related to the *original* plant configuration.

The model’s initial conditions were obtained through the measurements that were provided by the thermal imaging camera. As it can be noted in Figure 8, the thermal imaging camera is located upstream of phase 1. The second rolling mill pyrometer, which is located downstream of phase 6, provided the final temperature at the end of the whole rolling mill process. This temperature was compared with the estimate that was obtained so to validate the model. The purpose of the model is to be able to predict the value that was returned by the pyrometer that was downstream of stand 9, starting from the temperature value that was returned by the thermal imaging camera that was installed at the furnace outlet, and this was therefore, upstream of the whole process that has been taken into account.

For each tracked billet, a 3D representation has been created. A single initial thermal model with a time evolution factor has been designed for each tracked billet. The Stefan–Boltzmann constant has been considered, together with the mesh and the thermal properties. Additionally, scale-formation modelization has been considered, thereby, we suitably set the thermal conductivity parameters for the top layer of each billet. In fact, in phases 1–2, the presence of scale on the billet’s surface has to be taken into account [35,36].

In order to be capable to simulate the designed model, the problem of the estimation of the unknown parameters was solved. These parameters are involved in the heat transfer coefficients and the thermal heat flow computation. Some of these parameters are: the billet feed speed, the roll rotation speed, the temperature of the water in the phase of elimination of the scale, the temperature of the transport rollers, the final thickness of the billet at the end of the two rolling stages, the parameters relating to rolling stand rollers (the work-hardening exponent, the friction coefficient, the radius of the roller, the thermal conductivity, and the coefficient of resistance), the parameters relating to the rollers that transport the billets (the dimension of the billet area that is actually in contact with them and the thickness of the space vacuum that is generated between the billet and the rollers), and the duration of the single phases. The billet parameters are those which are contained in the database that is described in Section 2.2. In order to evaluate the goodness of the designed temperature decay model, for each considered billet, a percent relative error has been calculated as follows:

(1) $$error=\frac{T_{est}-T_{real}}{T_{real}}\cdot 100$$

where $T_{real}$ represents the scalar value that is associated to the measurements sequence that was provided by the second rolling mill pyrometer (stand 9) and $T_{est}$ represents the temperature estimation that was provided by the model in the same position. $T_{est}$ represents the average temperature that was recorded at the end of the rolling mill process on the top of the modelled billet, since the 3D model does not include the reduction of the thickness of the semi-finished product.

### 2.6. Computational Framework

A MATLAB environment has been used for the data preprocessing, data analysis, and to model the rolling mill stands billet temperature decay [37].

For the data preprocessing and data analysis, the MATLAB functions that are related to the scatter plot, determination index, and correlation coefficient computation were exploited.

For the modelization, the MATLAB Partial Differential Equation Toolbox has been used [37]. In particular, the Heat Transfer application has been exploited: functions for the solution of the heat transfer equations and the partial differential equations (PDE) through a finite element method were provided by the Toolbox.

The work has been executed using a laptop computer with the following specifications: Intel(R) Core(TM) i8-3840QM CPU with 3 GHz HDD.

## 3. Results and Discussion

This section reports the results and the discussion that are related to the billet data analysis and to the rolling mill stands billet temperature decay mathematical modelization.

In order to synthetize the results, the following convention will be adopted: TIC indicates the thermal imaging camera, FRMP indicates the first rolling mill pyrometer, and SRMP indicates the second rolling mill pyrometer.

### 3.1. Data Analysis Results (Original Plant Configuration)

As described theoretically in Section 2.4, the criteria that were initially adopted for billet data analysis were the creation of different billet clusters that were based on whether the billets had: the same production day, the same reheating group, the same thermal imaging camera temperature range, or the same diameter (of the related finished products). Table 4 reports example values of the determination indexes and of the correlation coefficients that are related to each of the considered sensors in the case of the cluster of billets that were produced on the same day. When one is observing the values that are shown in Table 4, no significant correlation can be inferred when one is using the production day as the partition criterion. This result was obtained for all of the previously cited partition criteria.

Nevertheless, in the majority of the analyzed diameter groups, the rolling mill pyrometers show, with respect to the measurements of the thermal imaging camera, better correlation properties with the rolling mill stands’ absorptionsof current. This result suggests that the positioning of the rolling mill pyrometers with respect to the thermal imaging camera’s position (in the *original* plant configuration) is more reliable.

As a consequence, the previously described criteria were discarded because of them not having satisfactory results. Good results were obtained with a fifth criterion, and as such, the sub-clustering of the previous criterion (the diameter of the finished products) was performed; the billets were further partitioned on the basis of their steel quality. The resulting clusters contain billets that were characterized by same quality as well as the same diameter. Table 5 reports example values of the determination indexes and of the correlation coefficients that are related to each of the considered sensors.

When one is observing the values that are shown in Table 5, a general improvement of the determination index and of the correlation coefficient can be observed. This result was obtained for all of the created groups according to the described partition criterion; this result applies to all of the sensors, but not to pairs that include the thermal imaging camera. Generally, the thermal imaging camera showed poor correlation properties. This fact can be due, for example, to the presence of scale: the thermal imaging camera, which was positioned at the furnace outlet and upstream of the descaler device, was affected by the scale formation on the billets.

**Figure 9:**
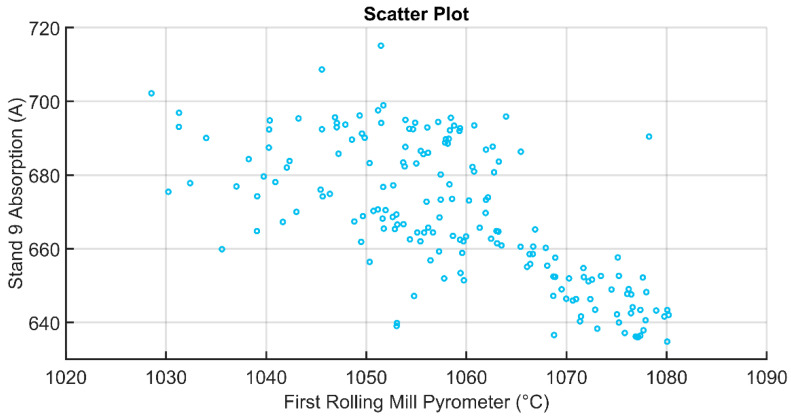
Scatter plot comparing the first rolling mill pyrometer and the absorption in stand 9.

**Figure 10:**
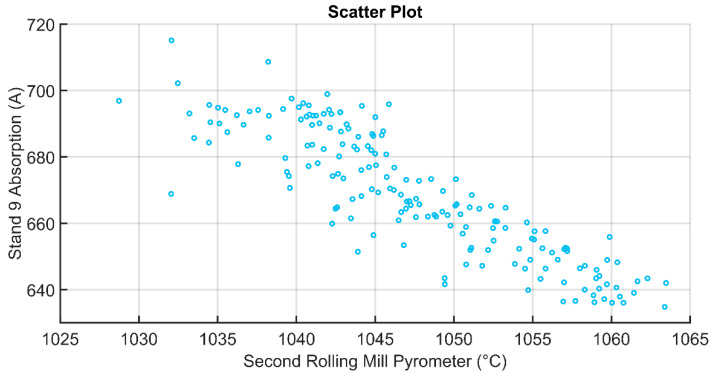
Scatter plot comparing the second rolling mill pyrometer and the absorption in stand 9.

Scatter plots that are related to the absorption in stand 9 and the first/second rolling mill pyrometers measurements are reported in Figure 9 and Figure 10, respectively. The observed inverse proportionality between the absorption in stand 9 and the first/second rolling mill pyrometers can be explained by the fact that a colder billet requires a major force (and major current absorption) to be plastically deformed in the rolling mill stands. So, the obtained results are in agreement with the physical laws of the considered process.

### 3.2. Data Analysis Results (Modified Plant Configuration)

The results and discussion that are reported in Section 3.1 suggested a modification of the position of the thermal imaging camera in order to improve thermal imaging camera data reliability. The *modified* plant configuration is reported in Figure 4. Note that, with respect to the original configuration (Figure 3 and Figure 8), the thermal imaging camera has been moved to a position that comes after the descaler in order to avoid the detrimental effects of scale formation on its measurements.

As a consequence of the new position of the thermal imaging camera, the same steps that are described in the Section 2.2, Section 2.3 and Section 2.4 have been followed (also, the billet tracking system has been updated).

**Figure 11:**
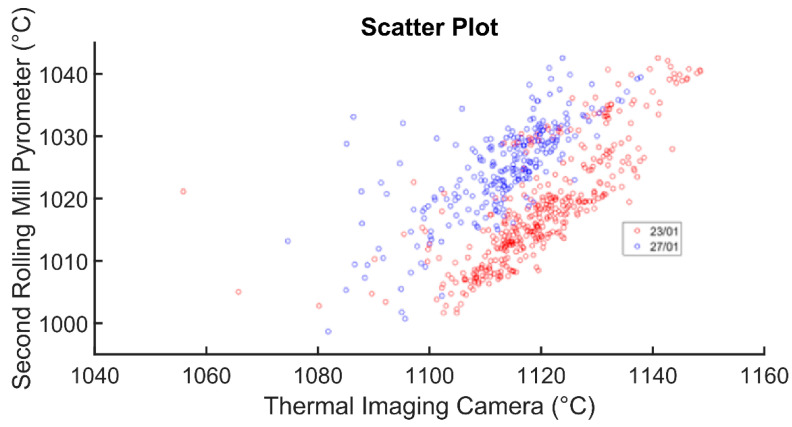
Scatter plot comparing the thermal imaging camera and the second rolling mill pyrometer.

Different production days have been considered, adopting the clustering methods that are described in Section 3.1. Table 6 reports the determination indexes and the correlation coefficients of each considered example (billets with the same quality of the steel and diameter of the related finished products) which are related to the thermal imaging camera/second rolling mill pyrometer pair. Figure 11 shows a scatter plot where two of the examples that are reported in Table 6 are shown. As expected, a directly proportional relationship is observed in Figure 11. Table 7 reports the same type of analysis that Table 6 does, but some of the clusters are merged.

The results that are shown in Table 6 and Figure 11 prove that the repositioning of the thermal imaging camera facilitated an improvement of the correlation and determination indicators that are related to the thermal imaging camera/second rolling mill pyrometer pair. Very satisfactory results are obtained only with the best clustering method that is in the Section 3.1: each cluster is characterized by billets of the same steel quality that are subjected to the same rolling mill processing (thereby, producing the same diameter for the finished products). By merging some billet clusters, the correlation features decrease (see Table 7). From a process point of view, this result can be explained by the fact that, generally, billets that are subjected to different rolling mill treatments have different physical characteristics. This can justify the fact that by aggregating billets belonging to more than one production cluster, the measurements of the two sensors become uncorrelated.

### 3.3. Modelization Results

**Figure 12:**
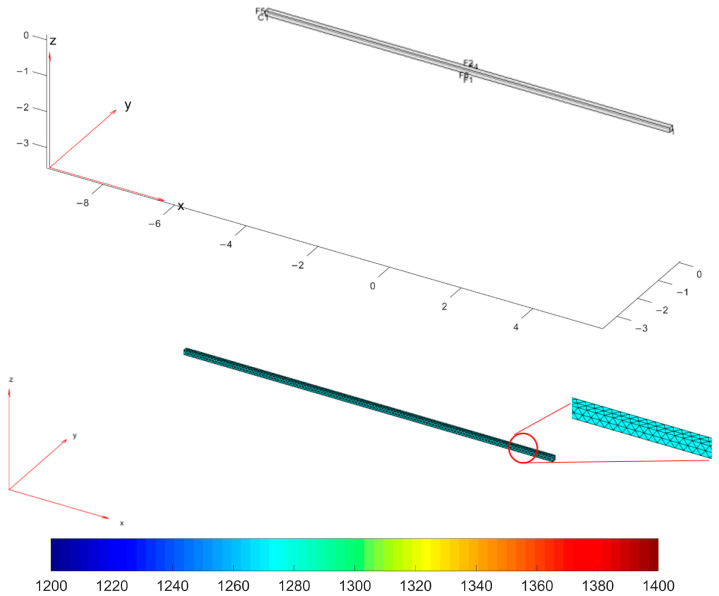
Modelization: 3D model of a billet, 3D model mesh, and color temperature scaling (K).

The mathematical modelization of the billet temperature decay within the rolling mill stands was based on a 3D representation of the billets, the meshing techniques that were used, and appropriate color temperature scaling. The modelization takes into account the *original* plant configuration. Figure 12 reports these modelization features.

**Figure 13:**
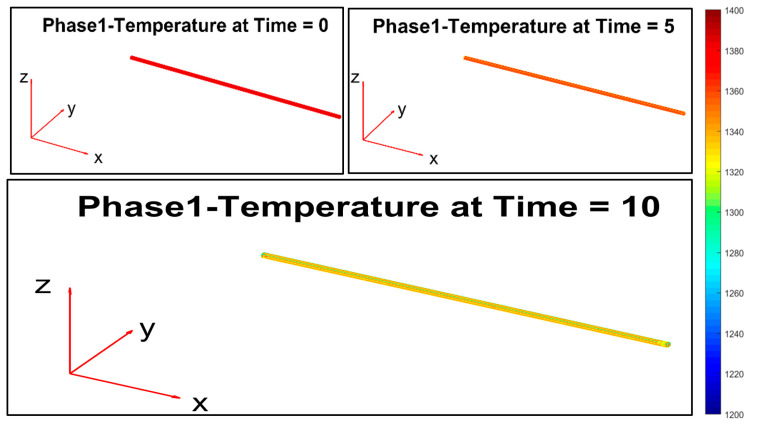
Modelization: phase 1 (movement between the thermal imaging camera and the descaler).

**Figure 14:**
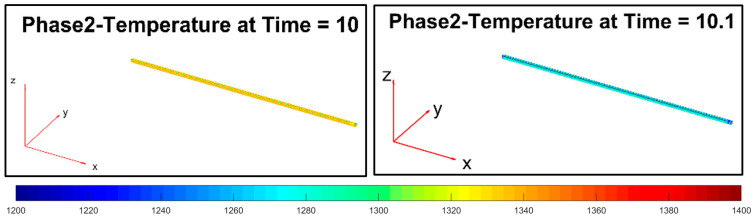
Modelization: phase 2 (descaler processing).

**Figure 15:**
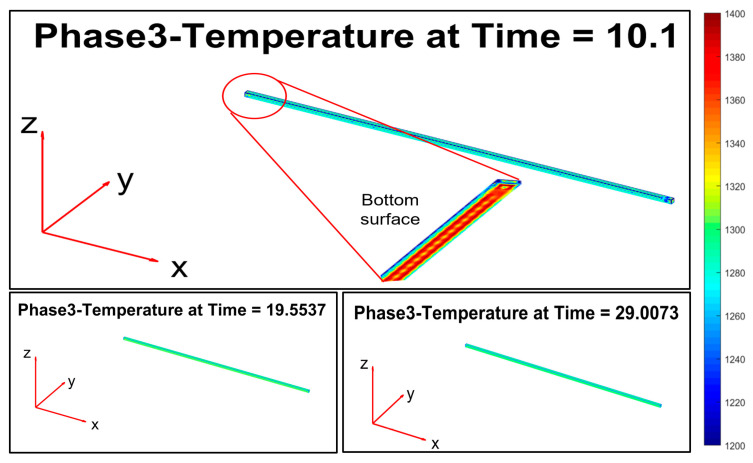
Modelization: phase 3 (movement between the descaler and stand 1).

**Figure 16:**
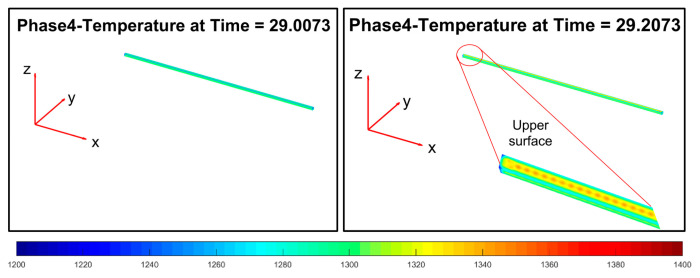
Modelization: phase 4 (processing between rolling mill stands 1–2).

**Figure 17:**
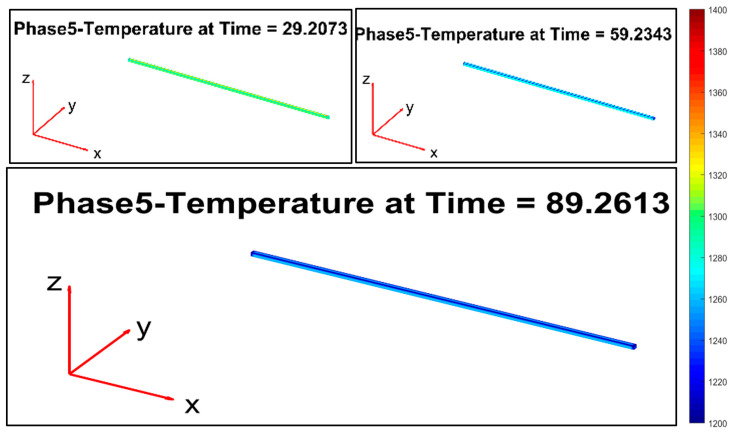
Modelization: phase 5 (movement between stand 2 and stand 3).

**Figure 18:**
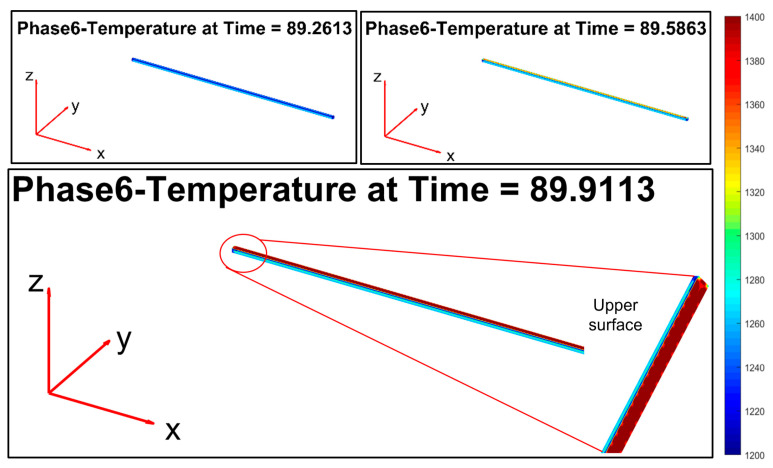
Modelization: phase 6 (processing between stand 3 and stand 9).

Figure 13, Figure 14, Figure 15, Figure 16, Figure 17 and Figure 18 report the simulation results that are related to each of the six steps of the modelization (the temperature in the model is expressed by K, while time is in seconds): the final state of each phase is the initial state of the subsequent phase. The simulation refers to a billet with the following characteristics:

- Length: 11.9 m;
- Section: 0.14 m × 0.14 m;
- Mass: 1830 kg;
- Rolling mill ambient temperature: 18.9 °C;
- Thermal camera temperature: 1108 °C;
- Rolling mill phase duration: 89.911 s;
- Second rolling pyrometer temperature: 1034 °C.

These data refer to a billet that was tracked through the designed billet tracking system.

During the first phase (Figure 13), the temperature that is related to the billet layers decreases due to radiation and natural convection heat transfer phenomena with the ambient air. The temperature of the lower layer of the billet is greater than the others due to the heat flow that is generated by the transport rollers.

In the second phase (Figure 14), a forced convection phenomenon between the billet and the descaler device’s water jet takes place (scale removal). The scale removal is simulated only on the top and side layers of the billet. This simulation choice was motivated by the fact that the bottom layers of the billet are in direct contact with the transport rollers and in this way, a negligible scale formation is expected.

During the third phase (Figure 15), initially, an equalization of the billet temperature takes place between the different billet layers. A bottom-up heat flow triggers a temperature increase on the top and side layers of the billet. Subsequently, the billet temperature continues to decrease due to the phenomena of heat transfer by radiation and natural convection with the ambient air. The temperature of the lower layer of the billet is higher than the others due to the heat flow that is generated by the transport rollers.

Phase 4 (Figure 16) introduces an increase of the temperature on the top layer of the billet, thanks to the simulated significant friction with the rolling mill stands.

Phase 5 (Figure 17) replicates phase 1, and the billet temperature continues to decrease.

Phase 6 (Figure 18) replicates phase 4: the second group of rolling mill stands cause a significant friction that triggers a temperature increase on the top layer of the billet.

At the end of the simulation, the mean temperature that is related to the top layer of the billet that is calculated by the model is 1042 °C, while the real temperature is 1034 °C. The relative error (see Equation (1)) is equal to 0.773%.

The data analysis (see Section 3.1) that was performed on the entire dataset was essential for providing a meaningful data subset for its use in a model evaluation: the clusters with poor a correlation-determination analysis were discarded.

## 4. Conclusions

This study aimed to address some aspects of the data analysis and modeling research in a steel industry, focusing on the billet preheating stage and the subsequent rolling mill stage.

An in-depth study of billet temperature sensors measurements (via a thermal imaging camera and rolling mill pyrometers) and of the current absorption values of the rolling mill stands was conducted, and as a result, we deduced that for the achievement of satisfactory correlation results, it is essential to group the billets that are characterized by the same type of steel and the same type of final product.

The data analysis was essential for providing a meaningful data subset, which was characterized by reliable correlation indexes, for its use in a model evaluation. By exploiting billet clusters that are characterized by satisfactory correlation properties, a mathematical model of the temperature decay that was related to the path that the billets took after exiting the reheating furnace was proposed.

The different initial temperatures in the rolling phase, the presence of possible stops during this phase, and the variability of the geometric and physical characteristics of the billets significantly affected the surface temperature of the billets, as these are characteristics that are closely related to the dynamic behavior of the heat transfer processes that occurred. The calibration of the designed temperature decay model took into account all of these parameters. The modelling results show very low relative percentage errors.

The conducted data analysis also showed that scale formation severely affects the thermal imaging camera measurements. The thermal imaging camera was moved to a position that was situated after the descaler device in order to enhance the correlation and determination indexes between the thermal imaging camera and the rolling mill pyrometers that were located downstream of the last rolling mill stand.

Future work will be focused on the modelization of the billet temperature decay in the rolling mill, exploiting data that are related to the updated thermal imaging camera’s location. Furthermore, a more advanced and in-depth data analysis on the billets could be executed, e.g., considering the reheating features of each billet within the reheating furnace. In addition, the results of the present work with the updated sensor configuration and the implementation of the proposed model can help with the management of constraints within an advanced process control system that is based on a model predictive control strategy [1].

## Figures and Tables

**Table 1:** Furnace zone lengths.

Furnace Zone	Length
Dead Zone	5.4 m
Pre-Heating Zone	4.1 m
Heating Zone	6.1 m
Soaking Zone	3.4 m

**Table 2:** Thermal imaging camera’s and pyrometers’ measuring range.

Sensor	Measuring Range
Furnace Inlet Pyrometer	0–500 °C
Thermal Imaging Camera	675–1800 °C (1 kHz)
First Rolling Mill Pyrometer	700–1200 °C
Second Rolling Mill Pyrometer	700–1100 °C

**Table 3:** Data that were selected for the proposed study.

Signal	Subprocess
Zones Temperature	Reheating Furnace
Smoke Exchanger Temperature	Reheating Furnace
Furnace Inlet Pyrometer	Reheating Furnace
Furnace Inlet Photocells	Reheating Furnace
Billet Charging Trigger	Reheating Furnace
Iron Wire Tie Counter	Reheating Furnace
Furnace Step Forward Triggers	Reheating Furnace
Furnace Step Backward Triggers	Reheating Furnace
Furnace Step Magnitudes	Reheating Furnace
Furnace Exit Photocell	Reheating Furnace
Billet Discharge Trigger	Reheating Furnace
Billet Re-Charging Trigger	Reheating Furnace
Thermal Imaging Camera	Reheating Furnace/Rolling Mill
Rolling Mill Inlet Photocell	Rolling Mill
Rolling Mill Stands’ Triggers	Rolling Mill
Rolling Mill Exit Photocell	Rolling Mill
First Rolling Mill Pyrometer	Rolling Mill
Second Rolling Mill Pyrometer	Rolling Mill
Ambient Temperature	Rolling Mill
Rolling Mill Stands’ Absorption	Rolling Mill
Billet Geometry	-

**Table 4:** Data analysis results (*original* plant configuration) for the billets with the same production day.

Sensors	Determination Index	Correlation Coefficient
TIC–Stand 1 Abs.	0.1178	0.3432
TIC–Stand 2 Abs.	0.1031	0.3211
TIC–Stand 3 Abs.	0.0000045	0.0021
TIC–Stand 4 Abs.	0.0189	−0.1376
TIC–Stand 5 Abs.	0.0219	−0.1479
TIC–Stand 6 Abs.	0.0016	0.0397
TIC–Stand 7 Abs.	0.0038	−0.0616
TIC–Stand 8 Abs.	0.00028	0.0170
TIC–Stand 9 Abs.	0.0048	−0.0691
TIC–FRMP	0.0340	0.1844
TIC–SRMP	0.1942	0.4407
FRMP–SRMP	0.4627	0.6802
FRMP–Stand 1 Abs.	0.0877	−0.2961
FRMP–Stand 2 Abs.	0.0762	−0.2761
FRMP–Stand 3 Abs.	0.1055	−0.3249
FRMP–Stand 4 Abs.	0.0549	−0.2342
FRMP–Stand 5 Abs.	0.0381	−0.1953
FRMP–Stand 6 Abs.	0.0144	−0.1199
FRMP–Stand 7 Abs.	0.0364	−0.1908
FRMP–Stand 8 Abs.	0.0225	−0.1500
FRMP–Stand 9 Abs.	0.0211	−0.1453
SRMP–Stand 1 Abs.	0.000027	0.0052
SRMP–Stand 2 Abs.	0.00012	0.0109
SRMP–Stand 3 Abs.	0.0093	−0.0964
SRMP–Stand 4 Abs.	0.0159	−0.1262
SRMP–Stand 5 Abs.	0.0745	−0.2729
SRMP–Stand 6 Abs.	0.000012	−0.0035
SRMP–Stand 7 Abs.	0.0203	−0.1424
SRMP–Stand 8 Abs.	0.0019	−0.0441
SRMP–Stand 9 Abs.	0.0032	−0.0563

**Table 5:** Data analysis results (*original* plant configuration) for the billets of the same steel quality and of the same diameter for the related finished products.

Sensors	Determination Index	Correlation Coefficient
TIC–Stand 1 Abs.	0.4007	−0.6330
TIC–Stand 2 Abs.	0.4840	−0.6957
TIC–Stand 3 Abs.	0.7240	−0.8509
TIC–Stand 4 Abs.	0.4953	−0.7038
TIC–Stand 5 Abs.	0.5532	−0.7438
TIC–Stand 6 Abs.	0.5176	−0.7195
TIC–Stand 7 Abs.	0.6279	−0.7924
TIC–Stand 8 Abs.	0.5799	−0.7615
TIC–Stand 9 Abs.	0.5636	−0.7507
TIC–FRMP	0.6017	0.7757
TIC–SRMP	0.6034	0.7768
FRMP–SRMP	0.8385	0.9157
FRMP–Stand 1 Abs.	0.4752	−0.6893
FRMP–Stand 2 Abs.	0.6633	−0.8144
FRMP–Stand 3 Abs.	0.8213	−0.9063
FRMP–Stand 4 Abs.	0.6740	−0.8209
FRMP–Stand 5 Abs.	0.7266	−0.8524
FRMP–Stand 6 Abs.	0.6043	−0.7773
FRMP–Stand 7 Abs.	0.6763	−0.8223
FRMP–Stand 8 Abs.	0.8218	−0.9066
FRMP–Stand 9 Abs.	0.7673	−0.8759
SRMP–Stand 1 Abs.	0.4308	−0.6564
SRMP–Stand 2 Abs.	0.6360	−0.7975
SRMP–Stand 3 Abs.	0.7936	−0.8908
SRMP–Stand 4 Abs.	0.7340	−0.8567
SRMP–Stand 5 Abs.	0.6775	−0.8231
SRMP–Stand 6 Abs.	0.6291	−0.7932
SRMP–Stand 7 Abs.	0.7094	−0.8423
SRMP–Stand 8 Abs.	0.7775	−0.8818
SRMP–Stand 9 Abs.	0.7994	−0.8941

**Table 6:** Data analysis results (*modified* plant configuration) for the billets of the same steel quality and of the same diameter for the related finished products.

Sensors	Determination Index	Correlation Coefficient
TIC–SRMP (example 1)	0.896	0.9466
TIC–SRMP (example 2)	0.84	0.9165
TIC–SRMP (example 3)	0.75	0.8662
TIC–SRMP (example 4)	0.544	0.7376
TIC–SRMP (example 5)	0.5	0.7071
TIC–SRMP (example 6)	0.914	0.9560
TIC–SRMP (example 7)	0.773	0.8791
TIC–SRMP (example 8)	0.604	0.7771
TIC–SRMP (example 9)	0.542	0.7360

**Table 7:** Data analysis results (*modified* plant configuration) for the billets of different clusters of steel quality and of the same diameter for the related finished products.

Sensors	Determination Index	Correlation Coefficient
TIC–SRMP (example 10)	0.616	0.7850
TIC–SRMP (example 11)	0.496	0.7042
TIC–SRMP (example 12)	0.267	0.5164
TIC–SRMP (example 13)	0.355	0.5956

## Data Availability

Not applicable.
